# Supplementary material for: Aβ efflux impairment and inflammation linked to cerebrovascular accumulation of amyloid-forming amylin secreted from pancreas
Source: Commun Biol. 2023 Jan 3;6:2. doi: 10.1038/s42003-022-04398-2 (PMC9810597; doi:10.1038/s42003-022-04398-2)
Supplement: Supplementary file 2 — Supplementary Information [file 42003_2022_4398_MOESM2_ESM.pdf]

Supplemental Figures and Figure Legends

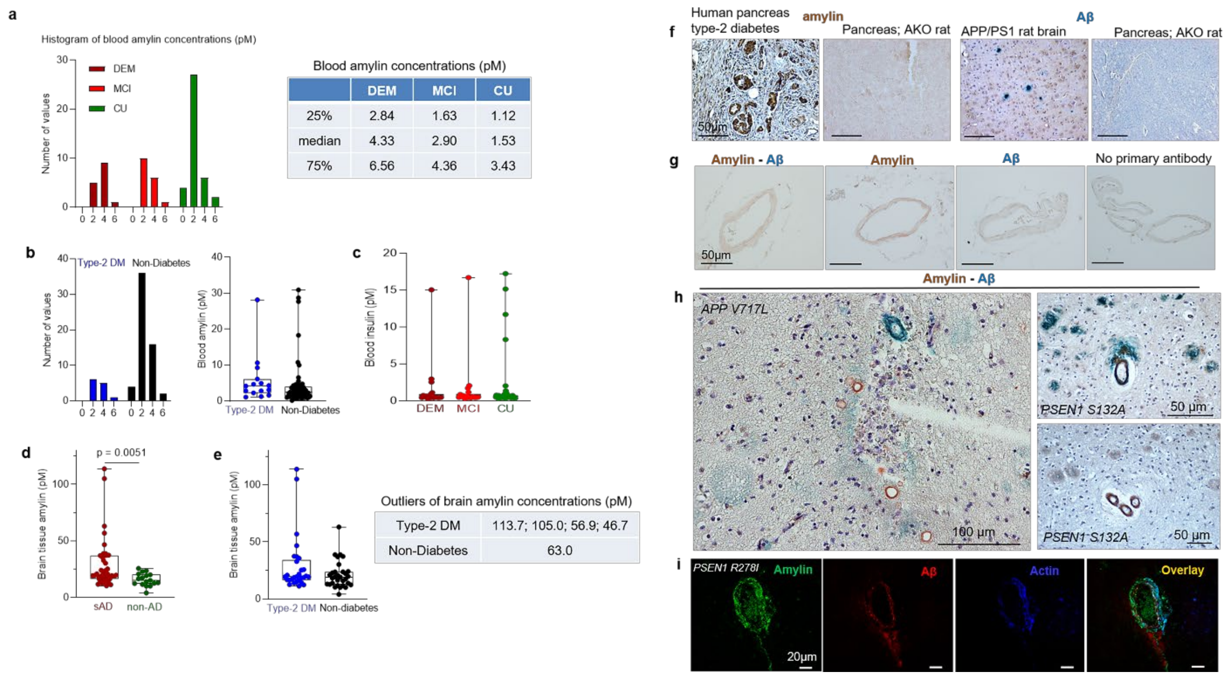

Supplemental Fig. S1 (A) Histogram of blood amylin concentrations, descriptive statistics and potential outliers in DEM, MCI and CU groups similar to those in Fig. 1C. (**B**) Same as in (A) in groups based on type-2 DM status (type-2 DM, n = 15 and Non-Diabetes, n = 65). **(C)** Blood insulin concentrations in DEM, MCI and CU groups similar to those in Fig. 1C. (**D**) Brain tissue amylin concentrations in persons with sAD ($n=42$) vs. those without AD ($n=18$). **(E)** same as in above in groups based on type-2 diabetes (type-2 DM; $n=29$) vs. non-diabetes (non-diabetes; $n=31$) status. **(F)** IHC analysis using an anti-amylin antibody on human pancreatic tissue from a person with type-2 diabetes (positive control for amylin deposition) and on pancreatic tissue from an AKO rat (negative control for amylin deposits). In IHC analysis using an anti-Aβ antibody, brain tissue from an APP/PS1 rat was the positive control for Aβ deposits, whereas pancreatic tissue from and AKO rat served as negative control for Aβ deposits. **(G)** IHC analyses of serial sections stained with anti-amylin, anti-Aβ, combined anti-amylin and anti-Aβ antibodies or only with secondary antibodies on temporal cortex tissue from an 86-years old cognitively unimpaired woman without type-2 diabetes; no amylin-Aβ cerebrovascular deposits were detected. **(H)** IHC analysis with anti-amylin (brown) and anti-Aβ (green) antibodies on brain sections from a fAD patient showing cerebrovascular amylin, Aβ and amylin-Aβ deposits in an old infarct area. Vascular amylin-Aβ deposits co-occurring with peri-arterial Aβ deposits and vascular amylin deposits without evidence of Aβ deposits are also included in the figure panel; see, Methods section for deconvolution and analysis of amylin and Aβ immunoreactive intensity signals. (**I**) Confocal microscopic analysis of a fAD brain section triple stained with anti-amylin

antibody (green), anti-Aβ antibody (red) and anti-α smooth muscle cell (SMC) actin antibody (blue). Data are presented as box and whiskers, non-parametric test**.**

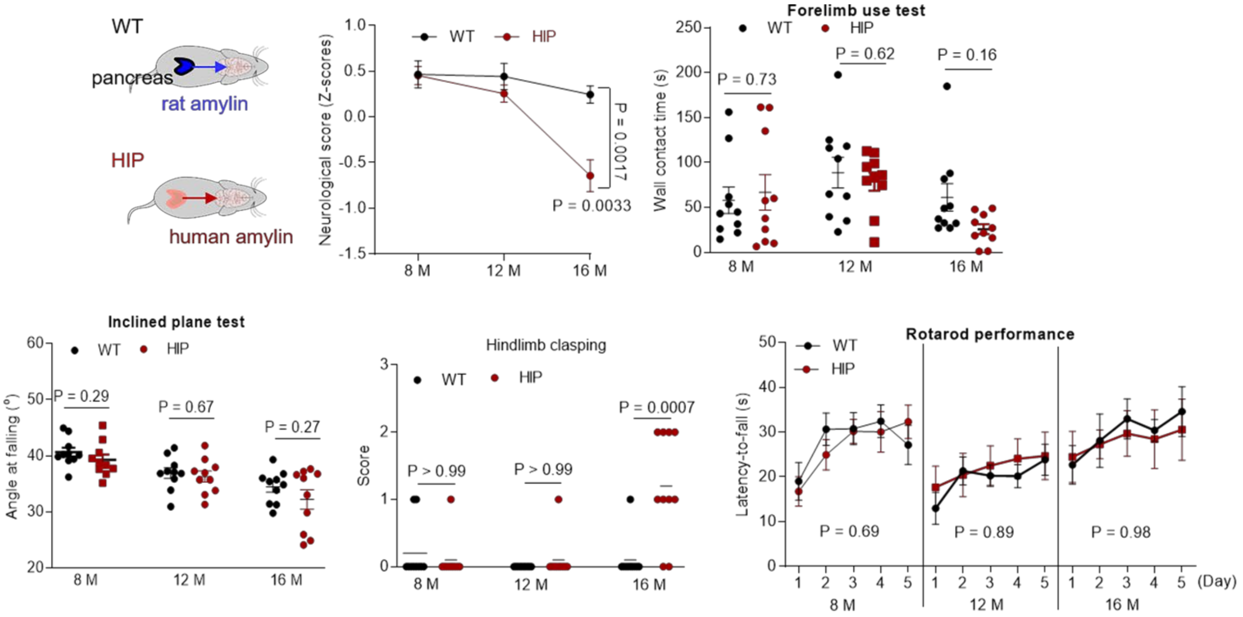

Supplemental Fig. S2 Average *z*-scores of behavior tests for motor function (forelimb use, balance ability on the inclined plane, hind limb clasping and Rotarod test) in HIP vs WT rats (*n* = 10 males/group) at 8 months (8M), 12 months (12M) and 16 months (16M) of age. Data are means ± SEM; 2-way ANOVA; 1-way ANOVA; non-parametric t-test.

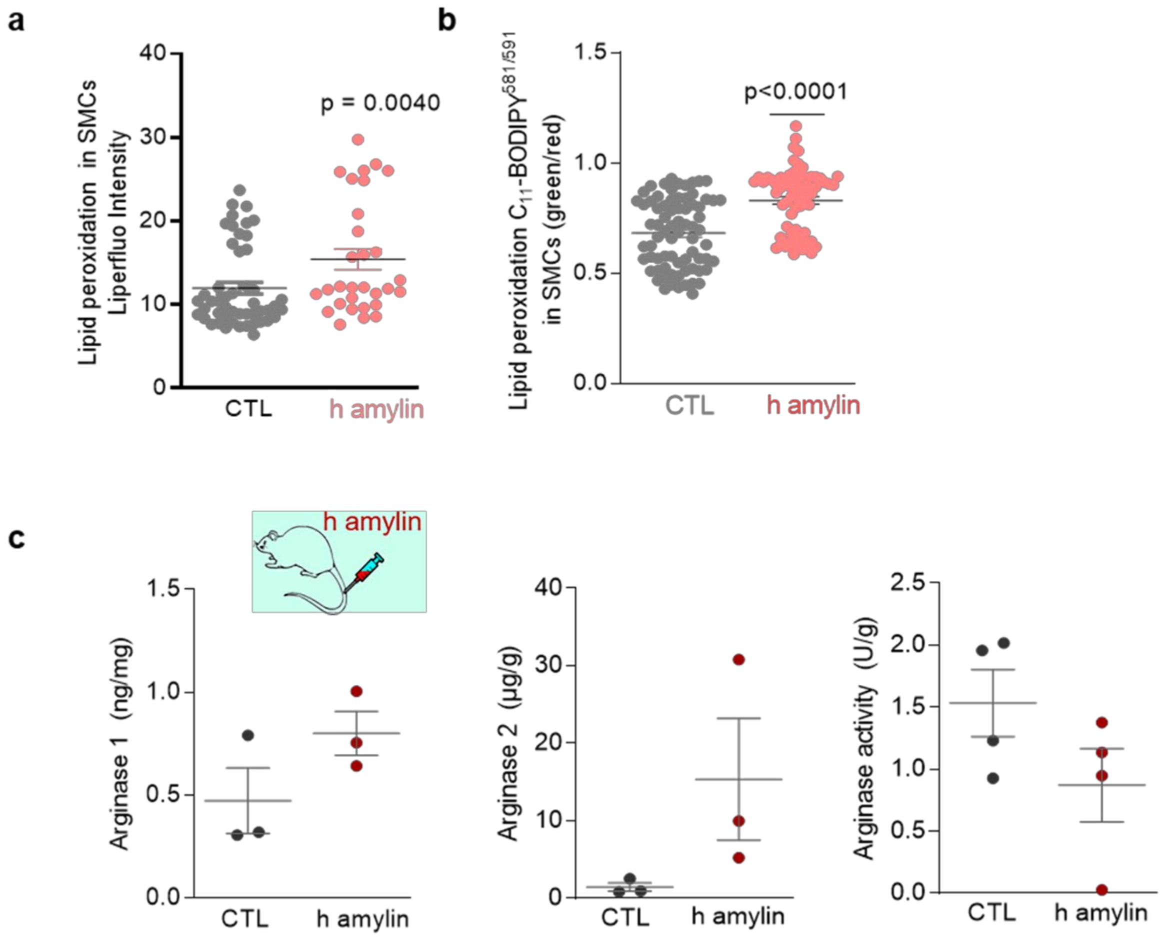

Supplemental Fig. S3 (A-B) Lipid peroxidation in pial artery SMCs from WT rats with/without incubation with 50 µM human amylin for 2 hours. Measurements were performed with Liperfluo **(B;** n>30 SMC from 3 WT rats**)** and C_{11}-BODIPY$^{581/591}$ **(C;** n>73 SMC from 5 WT rats**)**. Mann-Whitney non-parametric test. **(C)** Arginase activity, arginase-1, and arginase-2 protein levels in brain microvessel lysates from AKO rats intravenously injected with amyloid-forming human amylin at a concentration (60 µg/kg body weight; daily IV injection via tail vein for 1 week) (n=3 males/group, age 9-10 months). Data are means ± SEM; non-parametric t-test.

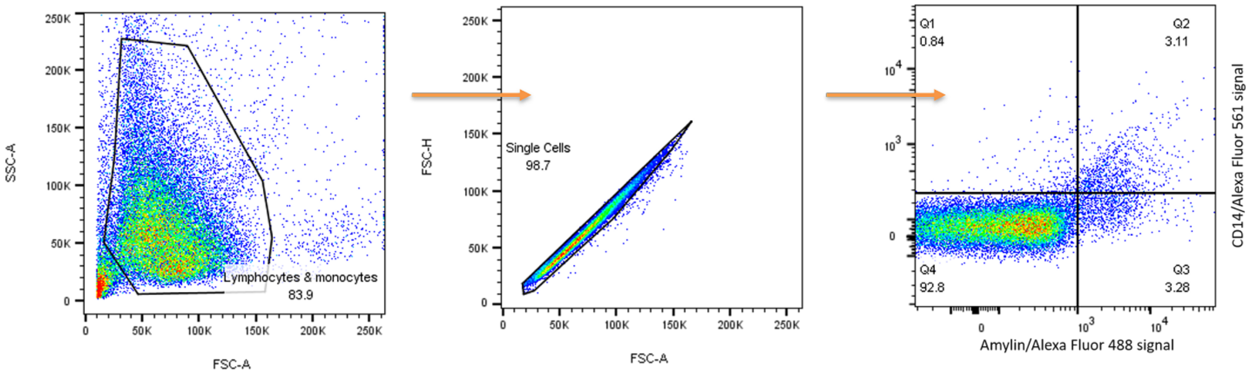

Supplemental Fig. S4. Gating strategy for monocytes positive for amylin. Gating shows side scatter (SSC) vs. forward scatter (FSC) to determine viable cell population (far left panel), the next gate FSC vs FSC is used to determine singlets within the viable cell population. These cells are then used to determine CD14^{+} monocytes vs amylin^{+} to determine the percentage of cells are Cd14^{+}Amylin^{+}.

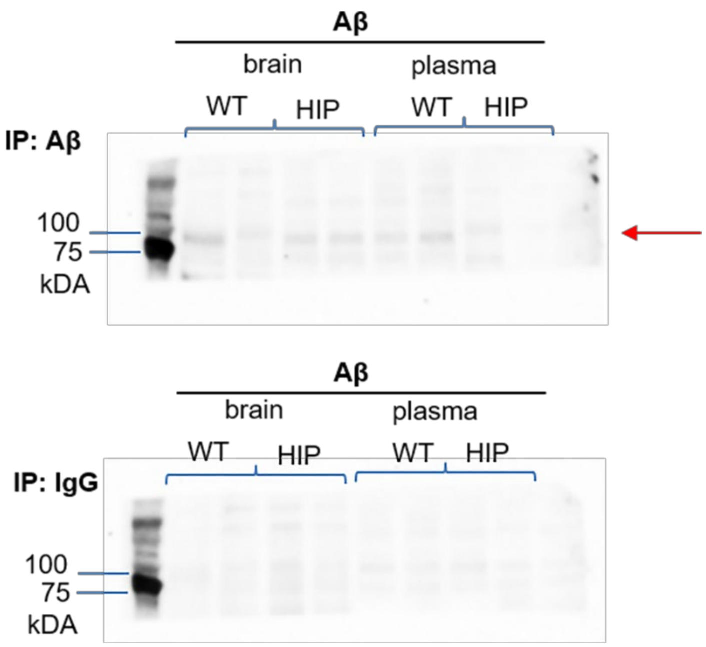

Supplemental Fig. S5. Negative control experiments to test the specificity of the bands identified in Western blotting after immunoprecipitation. Duplicate brain homogenate and plasma samples were used for immunoprecipitation with: 1, anti-Aβ antibody; and 2, anti-IgG antibody. After blotting and blocking, both membranes were incubated with the anti-Aβ antibody and further analyzed and imaged together. The results show no or minor immunoreactivity signal intensities in the IgG-immunoprecipitated sample set demonstrating the specificity of the Aβ antibody. Arrow points to predicted Aβ MW band in the Aβ enriched samples through immunoprecipitation (top panel).

Figure 5a (Upper panel)

Aβ

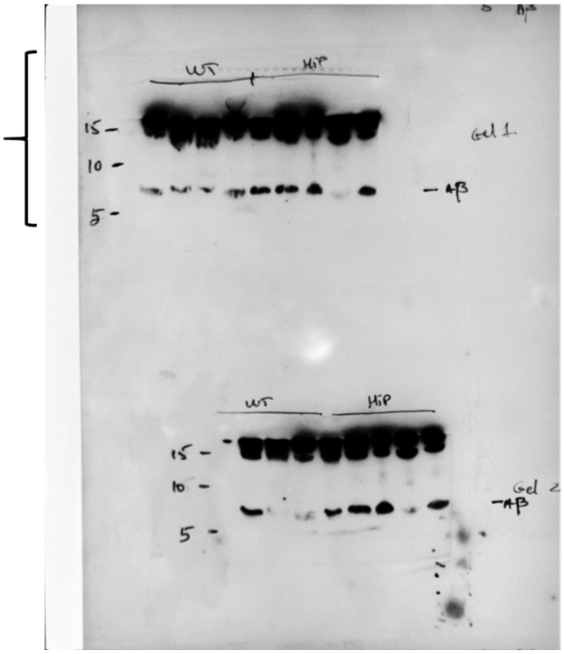

β-actin

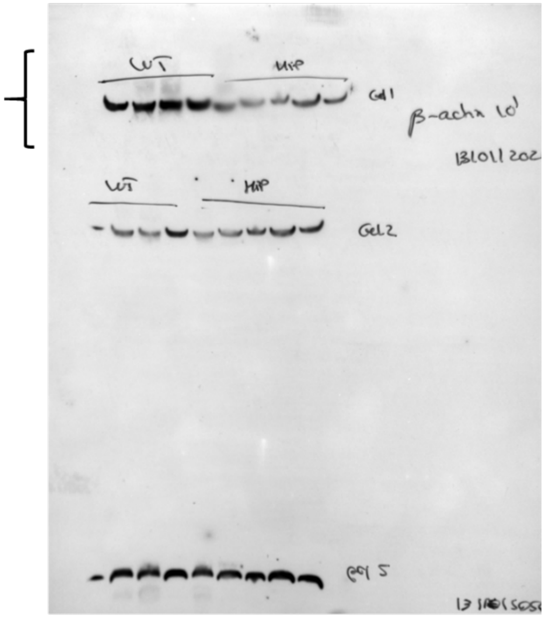

Figure 5a (Lower panel)

Aβ

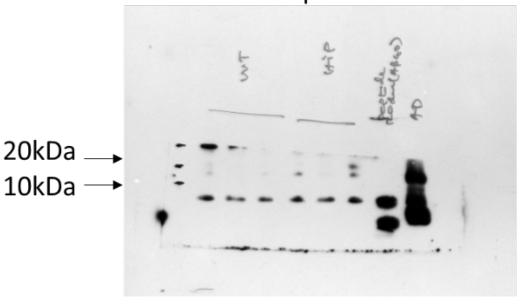

β-actin

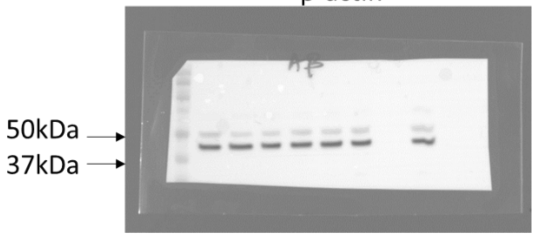

Figure 5b left panel (Plasma)

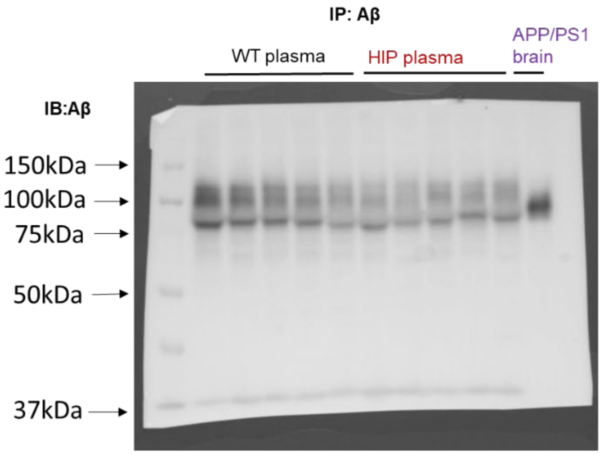

Figure 5b right panel (brain)

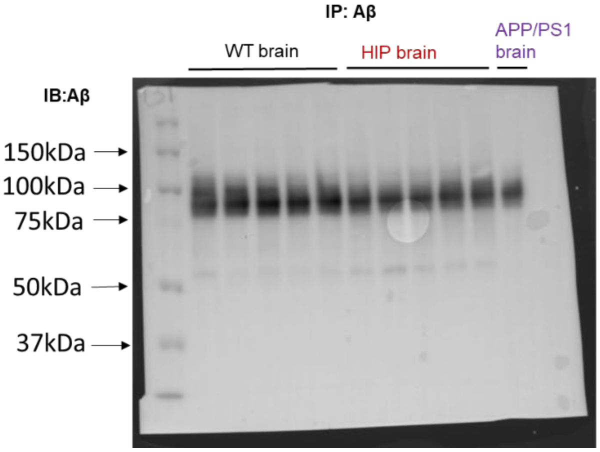

Figure 5g (upper panel)

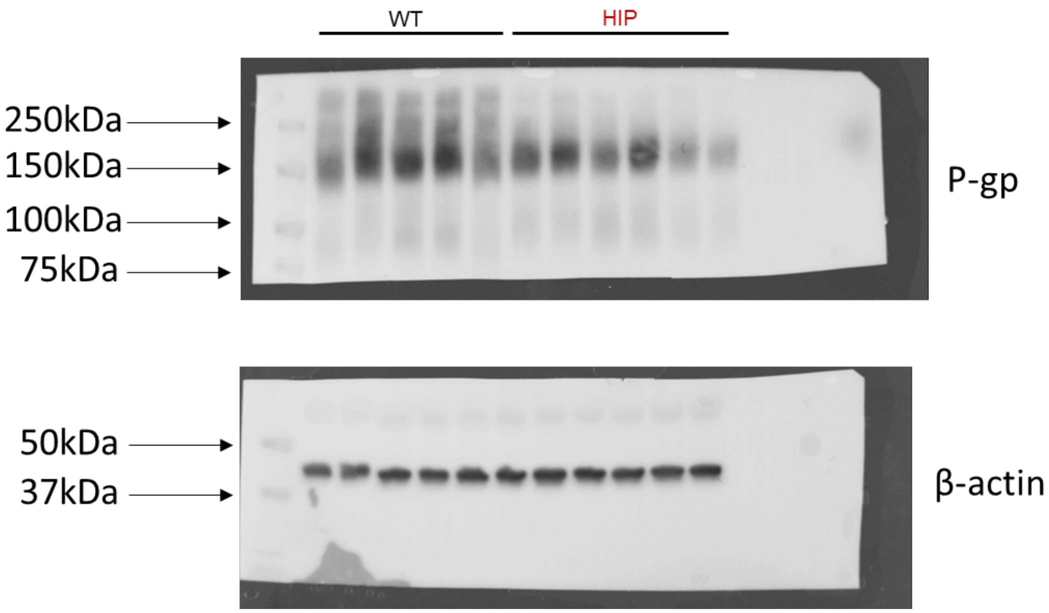

Figure 5g (lower panel)

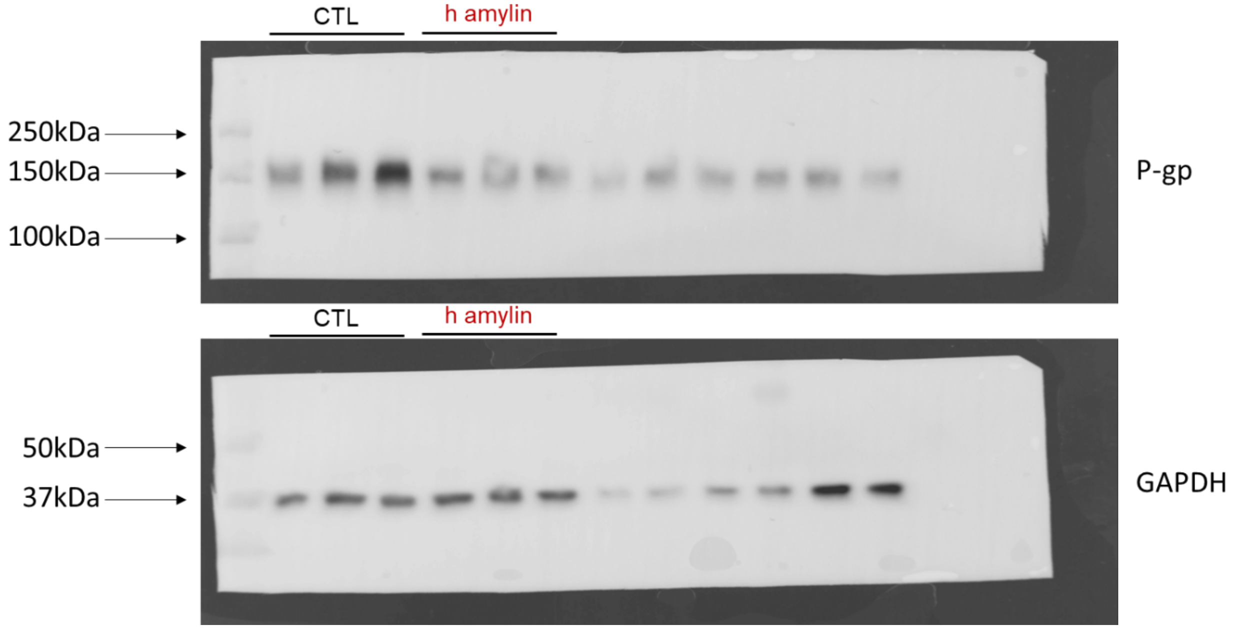

Figure 5h (upper panel)

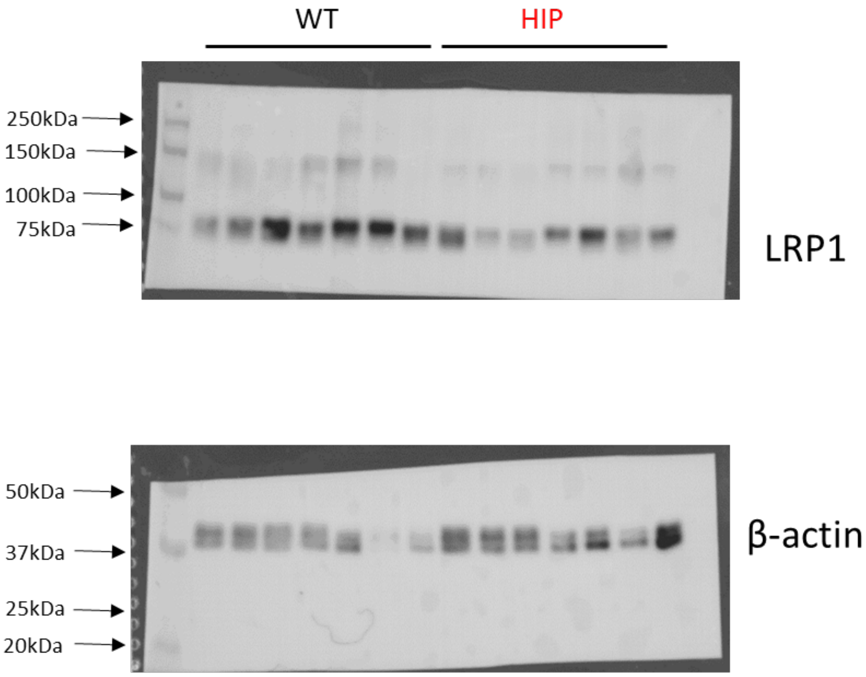

Figure 5h (lower panel)

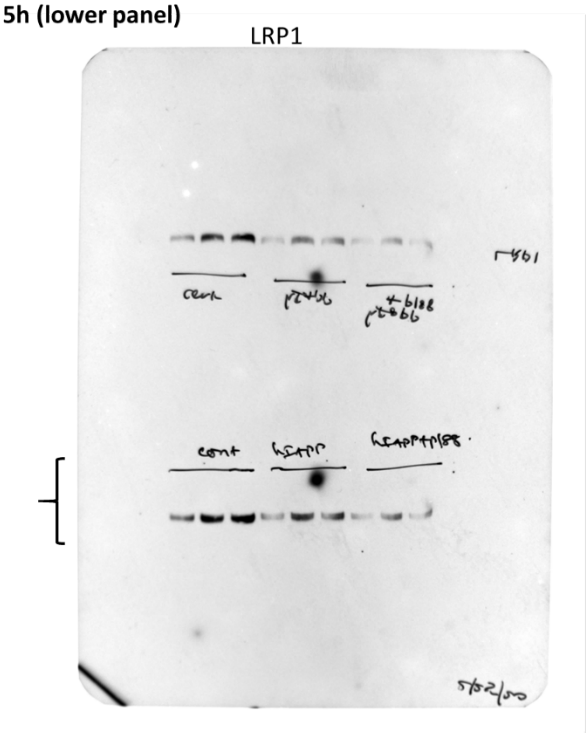

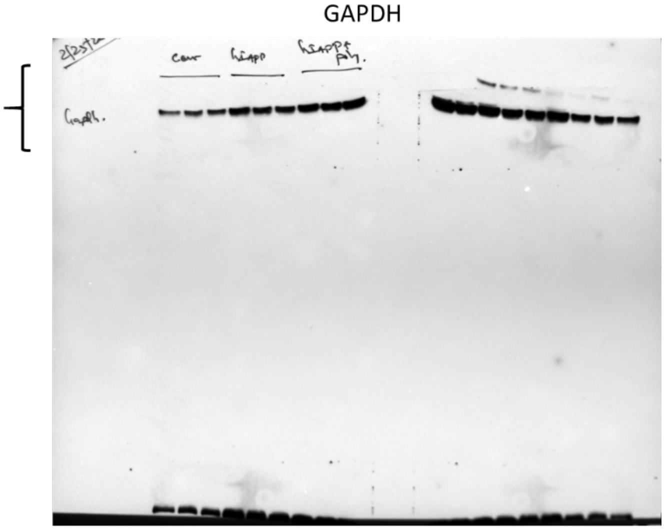

Fig 6c

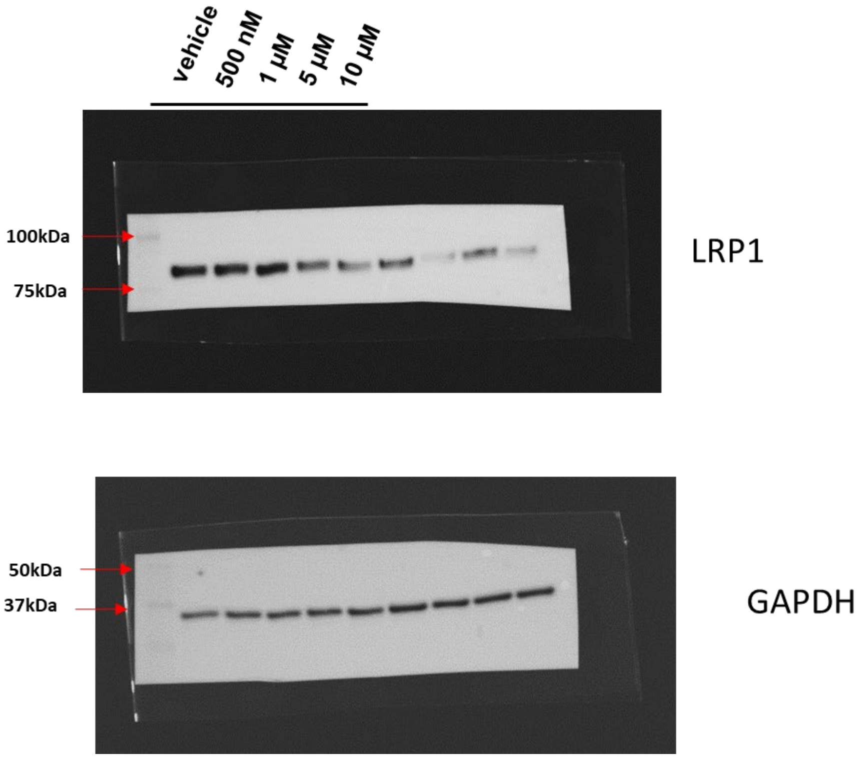

Fig 6k

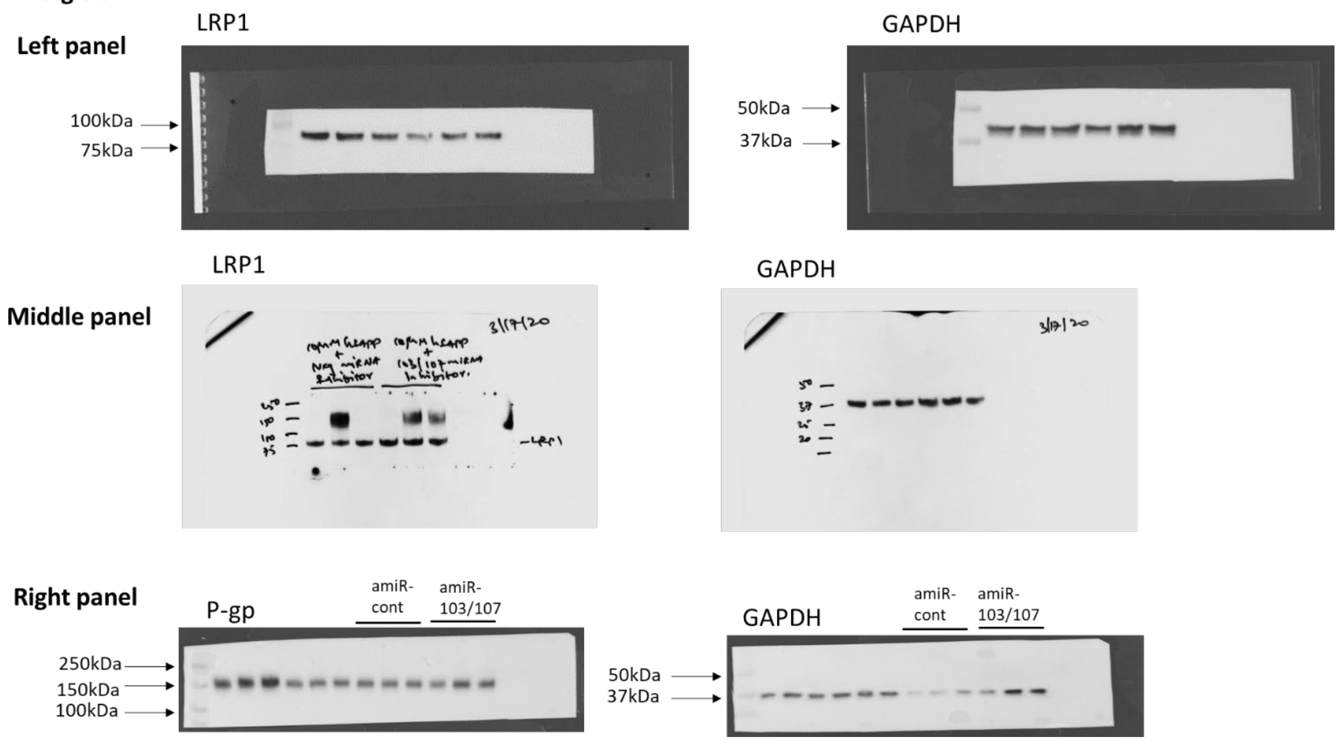

Supplemental Figure S5

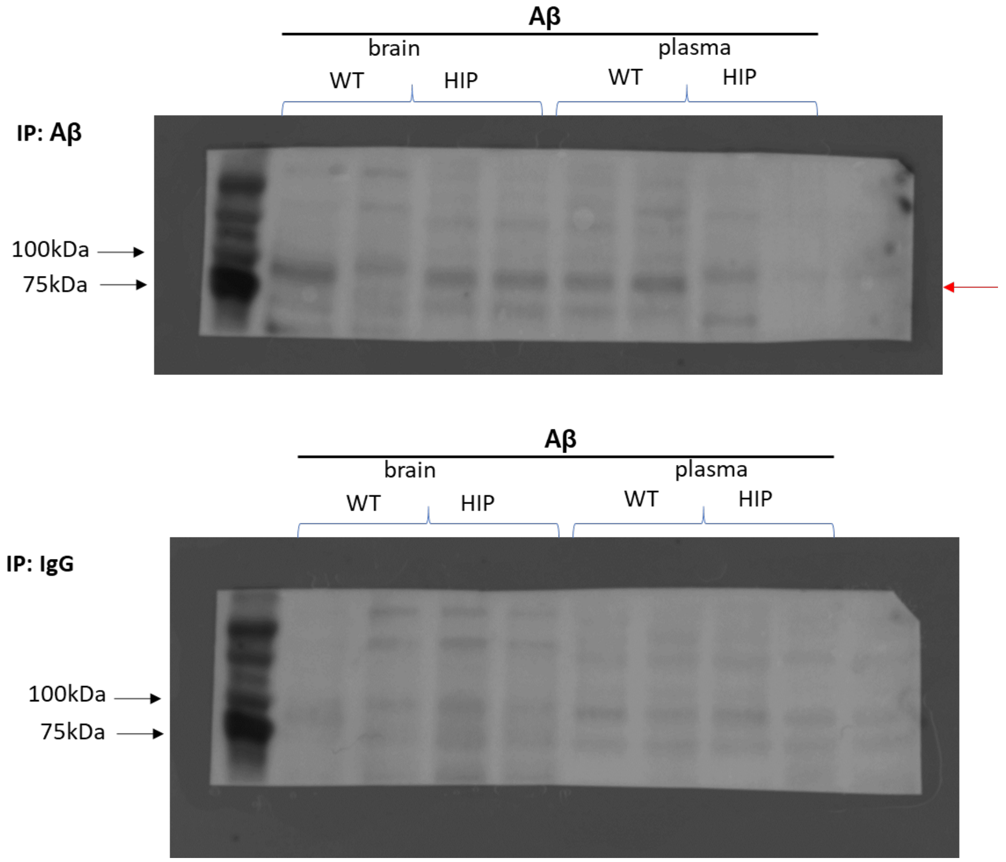

Supplemental Fig. S6. Negative control experiments to test the specificity of the bands identified in Western blotting after immunoprecipitation.
