# Supplementary material for: Aβ efflux impairment and inflammation linked to cerebrovascular accumulation of amyloid-forming amylin secreted from pancreas
Source: Commun Biol. 2023 Jan 3;6:2. doi: 10.1038/s42003-022-04398-2 (PMC9810597; doi:10.1038/s42003-022-04398-2)
Supplement: Supplementary file 3 — Description of Additional Supplementary Files [file 42003_2022_4398_MOESM3_ESM.pdf]

Description of Additional Supplementary Files

File name: Supplementary Data 1

Description: Source data for all the figures.
