# Supplementary material for: Aβ efflux impairment and inflammation linked to cerebrovascular accumulation of amyloid-forming amylin secreted from pancreas
Source: Commun Biol. 2023 Jan 3;6:2. doi: 10.1038/s42003-022-04398-2 (PMC9810597; doi:10.1038/s42003-022-04398-2)
Supplement: Supplementary file 5 — Reporting Summary [file 42003_2022_4398_MOESM5_ESM.pdf]

nature research

Corresponding author(s): Florin Despa; 2022/12/05

Reporting Summary

Nature Research wishes to improve the reproducibility of the work that we publish. This form provides structure for consistency and transparency in reporting. For further information on Nature Research policies, see Authors & Referees and the Editorial Policy Checklist.

Statistical parameters

When statistical analyses are reported, confirm that the following items are present in the relevant location (e.g. figure legend, table legend, main text, or Methods section).

n/a	Confirmed	
☐	☒	The exact sample size (*n*) for each experimental group/condition, given as a discrete number and unit of measurement
☐	☒	An indication of whether measurements were taken from distinct samples or whether the same sample was measured repeatedly
☐	☒	The statistical test(s) used AND whether they are one- or two-sided<br>*Only common tests should be described solely by name; describe more complex techniques in the Methods section.*
☒	☐	A description of all covariates tested
☐	☒	A description of any assumptions or corrections, such as tests of normality and adjustment for multiple comparisons
☐	☒	A full description of the statistics including central tendency (e.g. means) or other basic estimates (e.g. regression coefficient) AND variation (e.g. standard deviation) or associated estimates of uncertainty (e.g. confidence intervals)
☒	☐	For null hypothesis testing, the test statistic (e.g. *F*, *t*, *r*) with confidence intervals, effect sizes, degrees of freedom and *P* value noted<br>*Give P values as exact values whenever suitable.*
☒	☐	For Bayesian analysis, information on the choice of priors and Markov chain Monte Carlo settings
☒	☐	For hierarchical and complex designs, identification of the appropriate level for tests and full reporting of outcomes
☒	☐	Estimates of effect sizes (e.g. Cohen's *d*, Pearson's *r*), indicating how they were calculated
☐	☒	Clearly defined error bars<br>*State explicitly what error bars represent (e.g. SD, SE, CI)*

Our web collection on statistics for biologists may be useful.

Software and code

Policy information about availability of computer code

Data collection	No computer code was used
Data analysis	Graph Pad Prism 9.0

For manuscripts utilizing custom algorithms or software that are central to the research but not yet described in published literature, software must be made available to editors/reviewers upon request. We strongly encourage code deposition in a community repository (e.g. GitHub). See the Nature Research guidelines for submitting code & software for further information.

Data

Policy information about availability of data

All manuscripts must include a data availability statement. This statement should provide the following information, where applicable:

- Accession codes, unique identifiers, or web links for publicly available datasets
- A list of figures that have associated raw data
- A description of any restrictions on data availability

Data supporting the findings of this study are available in within the article and its supplementary information files. RNAseq data discussed in this publication have been deposited in the NCBI's Gene Expression Omnibus and are accessible through GEO Series accession number GSE221450 (https://www.ncbi.nlm.nih.gov/geo/query/acc.cgi?acc=GSE221450)

Field-specific reporting

Please select the best fit for your research. If you are not sure, read the appropriate sections before making your selection.

☒ Life sciences ☐ Behavioural & social sciences ☐ Ecological, evolutionary & environmental sciences

For a reference copy of the document with all sections, see nature.com/authors/policies/ReportingSummary-flat.pdf

Life sciences study design

All studies must disclose on these points even when the disclosure is negative.

Sample size	Sample size for animal behavior study was determined based on pilot study. Sample size was caluculated using Excel with known standard deviation, confidential interval of 95% and alpha equals 0.05.
Data exclusions	No data excluded
Replication	Replication was performed at different time point to verify the reproducibility of date obtained. The data was reproducible upon replication.
Randomization	Allocation of animals to the treatment groups and peptide injection were random.
Blinding	Observer-blind analyses were performed on all human tissue samples; the results were communicated to the AD Center at Univ of Kentucky to assess the relationships with AD pathology/cognitive function. Investigators were also blinded in the scoring of histological analyses and in the longitudinal animal behavior testing to prevent bias. Investigators were not blinded during pharmacological intervention/ injection because the interventions were performed by the same personnel. Investigators were not blinded during most of biochemical assays because it is not necessary. Data collection was performed at the same time for experimental groups with the same setting.

Reporting for specific materials, systems and methods

Materials & experimental systems

n/a	Involved in the study	
☒	☐	Unique biological materials
☐	☒	Antibodies
☐	☒	Eukaryotic cell lines
☒	☐	Palaeontology
☐	☒	Animals and other organisms
☒	☐	Human research participants

Methods

n/a	Involved in the study	
☒	☐	ChIP-seq
☐	☒	Flow cytometry
☐	☒	MRI-based neuroimaging

Antibodies

Antibodies used	Antibodies against amylin (1:200; T-4157, Bachem-Peninsula Laboratories), GFAP (1:400; 3670S, Cell Signaling Technology), CD68 (1:200; MCA341GA, Biorad,), CD11b (1:200; MCA275GA, Biorad,) and Aβ (1:400; clone 6E10, 803002, Biolegend) were the primary antibodies. Biotinylated IMPRESS horse anti-mouse-AP conjugated IgG (1:100, A3562, Sigma) and biotinylated anti-rabbit IgG (1:300, BA-1100, Vector) were the secondary antibodies.<br>In IHC, we used anti-amylin (1:200; clone E5; SC-377530; Santa Cruz, and 1:200; and T-4157, Bachem-Peninsula Laboratories), Aβ (1:400; clone 6E10, 803002, Biolegend), IL-1β (1:400; ab9722, Abcam), anti-collagen IV (1:500; ab6586; abcam), anti-alpha smooth muscle actin-Alexa Fluor 405 (1:200; ab210128, abcam), anti-caveolin-1 (1:100; sc-894; Santa Cruz, TX), anti-LRP1 (1:500; sc-57351; Santa Cruz), anti-4HNE (1:200; ab46545; abcam) were the primary antibodies. Secondary antibodies were: Alexa Fluor 488 anti-rabbit IgG (A11034; Thermo Fisher), Alexa Fluor 488 conjugated anti-mouse IgG (1:300; A11029; Invitrogen), Alexa Fluor 568 conjugated anti-rabbit IgG (1:200; A11036; Invitrogen), and Alexa Fluor 568 conjugated anti-mouse IgG (1:300; A11004; Invitrogen).
Validation	All of antibodies used in this manuscript are well-established by manufactures and other publications. We performed validation for amylin antibody in this study. Validation of amylin antibody was also established in previous publications cited in the manuscript. The publications are:<br>1. Ly, H. et al. Brain microvascular injury and white matter disease provoked by diabetes-associated hyperamylinemia. Ann Neurol 82, 208-222, doi:10.1002/ana.24992 (2017).<br>2. Jackson, K. et al. Amylin deposition in the brain: A second amyloid in Alzheimer disease? Annals of Neurology 74, 517-526, doi:10.1002/ana.23956 (2013).<br>3. Verma, N. et al. Intraneuronal Amylin Deposition, Peroxidative Membrane Injury and Increased IL-1beta Synthesis in Brains of

Alzheimer's Disease Patients with Type-2 Diabetes and in Diabetic HIP Rats. J Alzheimers Dis 53, 259-272, doi:10.3233/JAD-160047 (2016).

Eukaryotic cell lines

Policy information about cell lines

Cell line source(s)	primary rat brain microvascular endothelial cells (Cell Applications Inc)
Authentication	none of the cell lines were authenticated
Mycoplasma contamination	*Confirm that all cell lines tested negative for mycoplasma contamination OR describe the results of the testing for mycoplasma contamination OR declare that the cell lines were not tested for mycoplasma contamination.*
Commonly misidentified lines (See ICLAC register)	*Name any commonly misidentified cell lines used in the study and provide a rationale for their use.*

Animals and other organisms

Policy information about studies involving animals; ARRIVE guidelines recommended for reporting animal research

Laboratory animals	This study used transgenic rats: TgF344-19, provided by Charles River Laboratory; HIP rats provided by Charles River Laboratory and amylin knock-out rats generated by Transposagen and previously described (Ly, H. et al. Brain microvascular injury and white matter disease provoked by diabetes-associated hyperamylinemia. Ann. Neurol. 82, 208–222; 2017)
Wild animals	No wild animals were used in this study.
Field-collected samples	No field-collected samples were used in this study.

Flow Cytometry

Plots

Confirm that:

☒ The axis labels state the marker and fluorochrome used (e.g. CD4-FITC).

☒ The axis scales are clearly visible. Include numbers along axes only for bottom left plot of group (a 'group' is an analysis of identical markers).

☒ All plots are contour plots with outliers or pseudocolor plots.

☒ A numerical value for number of cells or percentage (with statistics) is provided.

Methodology

Sample preparation	Blood from both humans and rats (100 µl) was incubated with a mixture of the primary antibodies CD14-Alexa Fluor 561 (Abcam, ab203294, 1:400) and human amylin-Alexa Fluor 488 (Santa Cruz, SC-377530, 1:100) for 30 minutes at ambient temperature. The blood was lysed in 2ml of lysis buffer (Cat# 349202) for 5 minutes and washed with 1X PBS with 4% fetal calf serum (FCS). The remaining cells were then washed and incubated with secondary antibodies for 30 minutes at ambient temperature. They finally were washed again and resuspended in 0.5 ml of 1X PBS with 4% FCS.
Instrument	Cytometers BD Symphony A3
Software	Data was acquired using BDFacsDiva and analyzed using FlowJo v10 software.
Cell population abundance	The amount of viable cells in each sample varied, meaning the number of events taken varied for each sample. Cells within our live cell population were used to determine the fraction of our target cell population in each sample.
Gating strategy	Cells were gated on SSC-A vs FSC-A to determine live cell population. Doublets were gated out using FSC-H vs FSC-A. Cells were further gated to select human amylin on monocytes that were positive for both PE and BB515 (Q2 in figures). Negative control (no antibody) and positive control were used to set the upper and lower boundaries.

☒ Tick this box to confirm that a figure exemplifying the gating strategy is provided in the Supplementary Information.

Magnetic resonance imaging

Experimental design

Design type	T2-weighted anatomical MR scanning and pseudo-continuous arterial spin labeling (pCASL)
Design specifications	Live animals were scanned (n=10/group).

Behavioral performance measures | NA

Acquisition

Imaging type(s) | Structural images & pCASL

Field strength | 7 Tesla

Sequence & imaging parameters | Sequence type: gradient echo spacing 12.2 ms, echo trains per slice 39, RF pulse type low SAR.
Imaging type: T2-weighted anatomical image
Filed of view: 40 mm.
Matrix size: 256x192.
Slice thickness: 1 mm. Orientation: T> S-0.5; TR:3000ms/TE:24ms/Flip angle: 180 degree
Cerebral perfusion was assessed using pseudo-continuous arterial spin labeling (pCASL) technique Matlab scripts were written using Ze Wang's ASL Matlab toolbox3, which in turn use the SPM12 tool box (https://www.fil.ion.ucl.ac.uk/spm/). The scripts were run in batch mode. Briefly, the data was read in and converted to DICOM format. The anatomical and perfusion images were coregistered and filtered with the ASL toolbox routines. A mask was manually defined for each rat to exclude both tissue from outside the brain as the thresholding was sometimes insufficient to exclude those tissues. It was also determined that pixels at the edge of the brain introduced large variations perfusion values – presumably due to partial volume and susceptibility effects, and thus were also excluded. Perfusion values were determined without the knowledge of which groups the rats belonged to so as to avoid bias.
Orientation: T > S-0.5
TR: 3000 ms/TE: 24ms/Flip angle: 180 degree

Area of acquisition | Whole brain scan was used.

Diffusion MRI ☐ Used ☒ Not used

Preprocessing

Preprocessing software | Syngo MR B15 (Siemen). No data processing was performed.

Normalization | Prescan normalize was turned on.

Normalization template | NA

Noise and artifact removal | To avoid artifacts caused by unstable motion, respiratory rate was maintain 40-90 breaths per minute (resting stage).

Volume censoring | NA

Statistical modeling & inference

Model type and settings | NA

Effect(s) tested | NA

Specify type of analysis: ☒ Whole brain ☐ ROI-based ☐ Both

Statistic type for inference (See Eklund et al. 2016) | NA

Correction | NA

Models & analysis

n/a	Involved in the study
☒	☐ Functional and/or effective connectivity
☐	☒ Graph analysis
☒	☐ Multivariate modeling or predictive analysis

Graph analysis | Graph Pad Prism 9.0
